# Fabrication and Characterization of a Nanofast Cement for Dental Restorations

**DOI:** 10.1155/2021/7343147

**Published:** 2021-09-09

**Authors:** Kh. Yousefi, H. Danesh Manesh, A. R. Khalifeh, A. Gholami

**Affiliations:** ^1^Department of Materials Science and Engineering, School of Engineering, Shiraz University, 71348-51154 Shiraz, Iran; ^2^Department of Pharmaceutical Biotechnology, School of Pharmacy, Shiraz University of Medical Sciences, Shiraz, Iran

## Abstract

This study was aimed at fabricating and evaluating the physical and bioproperties of nanofast cement (NFC) as a replacement of the MTA. The cement particles were decreased in nanoscale, and zirconium oxide was used as a radiopacifier. The setting time and radiopacity were investigated according to ISO recommendations. Analysis of color, bioactivity, and cytotoxicity was performed using spectroscopy, simulated body fluid (SBF), and MTT assay. The setting time of cement pastes significantly dropped from 65 to 15 min when the particle sizes decreased from 2723 nm to 322 nm. Nanoparticles provide large surface areas and nucleation sites and thereby a higher hydration rate, so they reduced the setting time. Based on the resulting spectroscopy, the specimens did not exhibit clinically noticeable discoloration. Resistance to discoloration may be due to the resistance of zirconium oxide to decomposition. Scanning electron microscopy (SEM), X-ray diffraction (XRD), and infrared spectroscopy (FTIR) examinations of the immersed SBF samples showed apatite formation that was a reason for its suitable bioactivity. The results of cell culture revealed that NFC is nontoxic. This study showed that NFC was more beneficial than MTA in dental restorations.

## 1. Introduction

Calcium silicate-based cement named MTA was first developed as a root-end filling material, because of its clinical characteristics like bioactivity, biocompatibility, low solubility, adaptation to tooth structures, dimensional stability, and sealing ability [1–3]. MTA mainly consisted of dicalcium silicate, tricalcium silicate, tricalcium aluminate, and a small amount of bismuth oxide that has been added for radiopacity purposes. The limitations, such as poor handling quality, low strength, long setting time, discoloration, and considerable expense, make its use a restoration material complicated [4, 5].

Dental cements are chemically bonded materials that are bonded through chemical reactions without the use of a high-temperature process [6]. One of the most widely used cements is MTA. MTA is a hydrophilic cement powder that produces harden compounds when in contact with water. The process is named hardening or setting. A cement dental filler material should ideally have a relatively short setting time to avoid being washed away by saliva and reduce the possibility of the unset material irritating oral tissues. MTA has been shown to have a long setting time that confers many difficulties in handling during dental treatment processes [7]. The setting time for MTA in normal conditions is more than 2.5 to 3 hours [8], and in some treatments, more than one attempt is required to place a final restoration in a tooth. Researchers have made efforts to decrease the cement setting time using some additives and new formulations. For instance, several groups have made changes in the formulations of both the solid and liquid phases of MTA cement by adding calcium chloride, potassium chloride, or calcium formate to accelerate the setting time [9, 10]. Using these solutions as the hydration accelerant may enhance the biocompatibility but not compromise MTA's antibacterial and mechanical properties.

The aesthetic appearance of dental treatment is an essential issue for clinicians and patients, especially in the frontal part of the mouth. Hence, the color stability of endodontic materials is considered a critical factor for clinical success [11, 12]. MTA was first introduced in a grey form (GMTA) [13]. Discoloration has been reported as the primary problem connected to this dental material. After viewing the tooth discoloration caused by gray MTA, white MTA (WMTA) with reduced Al_2_O_3_, MgO, and FeO was developed with an off-white color to overcome this shortcoming [14]. However, several studies reported that tooth discoloration was induced even by WMTA. Marciano et al. reported that bismuth oxide's tangency with sodium hypochlorite (NaClO) leads to discoloration of ProRoot WMTA and suggested tangency should be avoided [15]. Many efforts have been carried out to find an alternative for bismuth oxide as a radiopacifier. Two products named ENDOCEM and Retro MTA were introduced by Kim et al. in which bismuth oxide has been replaced by zirconium oxide [16]. Although the producers claim that these sealer materials do not cause discoloration, there is no evidence to support their claim.

This research was aimed at developing a novel cement base dental material with a short setting time and resistance to discoloration. Additionally, the bioactivity, toxicity, and radiopacity of the acquired cement were examined.

## 2. Materials and Methods

### 2.1. Material

The cement powder used in this study mainly consists of dicalcium silicate, tricalcium silicate (Sanat Avaran Vista, Iran), and zirconia that was used as a radiopacifier. Absolute ethanol (molecular weight range 146,000–186,000 g/mol, degree of hydrolysis > 99.0%; Aldrich, St Louis, MO) was the solution. Particle size analysis (PSA) was performed on the cement powders utilizing VASCO Flex equipment (Cordouan Technologies, Pessac, France). The morphology of the prime powders was studied with field emission scanning electron microscopy (FESEM, MIRA3-TESCAN, Czech Republic).

### 2.2. Fabrication of Cement

The first step was the preparation of the NFC powders. To accomplish this, the cement particles were reduced to nanosize using Wet-Stirred-Media-Milling (WSMM). The WSMM is performed in a vertical cylindrical crushing chamber made of polyethylene. The ceramic beads and cement were added to the grinding chamber. Then, absolute ethanol (99.9%, Merck, Germany) was added to the mixture up to half the volume of the container. Afterward, the mixture was stirred for 15 hours using a stirrer. The beads were separated from the solution and mixture using a mesh. For obtaining dry cement, the ethanol was evaporated in a drying cabinet for 24 hours and at a temperature of 37°C. After the cement powder was dried, zirconia was added to the cement powder as a radiopacifier. This mixture was named NFC. The PSA method was utilized to measure final particle sizes. FESEM was used to observe the morphology of the prepared NFC powders.

### 2.3. Paste Preparation and Molding

**Figure 1:**
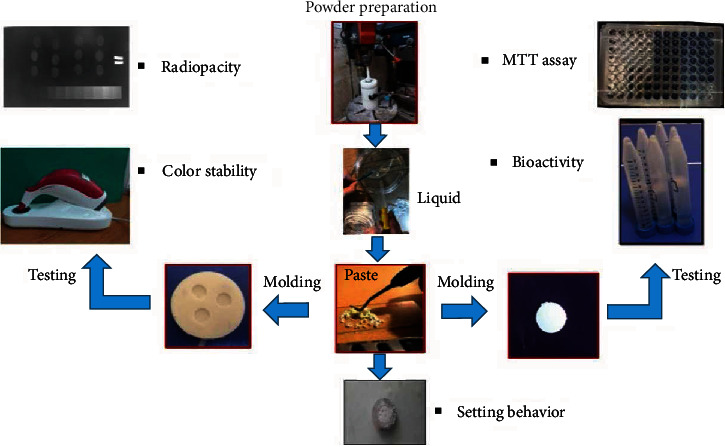
The material processing flow chart.

In this stage, the nanocement powders were mixed with distilled water, and the mixture was gently stirred using a spatula for 30 seconds to attain a homogenous and deformable paste. The prepared paste was used to study the setting behavior. Afterward, the obtained pastes were molded in silicone molds and allowed to set for color stability, bioactivity, toxicity, and radiopacity examinations. The material processing is schematically shown in Figure 1.

### 2.4. Setting Evaluation

The NFC setting behavior was investigated according to ISO 6876/2012 [17]. The freshly prepared cement pastes were molded in a stainless steel mold. Then, the samples were incubated under a temperature of 37°C and about 95% relative humidity. Afterward, a flattened indenter with 1 mm diameter and a 400 g load was perpendicularly dipped into the paste surface. The process was repeated every 60 seconds. The final setting time was recorded when the indenter was not capable of producing an indentation.

### 2.5. Evaluation of Color Stability

One of the aims of this research was to investigate the color stability of the NFC. To do so, the prepared cement pastes were formed in a cylindrical silicon mold 10 mm in diameter and 2 mm high. The setting time was 24 hours at 100% humidity and 37°C. The cement samples were then dried and tested. Afterward, the samples were immersed in color stability solutions for 24 hours. The solutions included water (H_2_O), sodium hypochlorite solution (NaClO), and hydrogen peroxide (H_2_O_2_).

Digital images of the cement samples were also taken before and after soaking by a digital camera (Canon, EOS 4000D, Japan). Analysis of color was performed using a spectrometer (VITA Easyshade Advance 4.0, Germany). Measurement was repeated three times by the same operator under steady laboratory light. The equipment was calibrated before each measurement to obtain more accurate results. The difference in color was calculated by the Commission International Del'eclairage (CIE) system and utilizing the following formula [18]:

(1) $$\begin{array}{c} \Delta E={\left(\frac{{\left(\Delta l\right)}^{2}+{\left(\Delta a\right)}^{2}+{\left(\Delta b\right)}^{2}}{2}\right)}^{1/2}, \end{array}$$

where Δ*E* is the color difference before and after immersion, ∆*l* shows the change in luminosity, ∆*a* shows the change in the red-green parameter, and ∆*b* shows the change in the yellow-blue parameter.

### 2.6. Bioactivity Property Evaluation

The bioactivity has been evaluated according to ISO 23317/2014 [19]. The samples of this examination were 10 mm in diameter and 2 mm in length. After setting, the prepared samples were immersed in simulated body fluid (SBF) for 1 and 14 days. SBF is a solution with an ion composition close to that of the human blood plasma buffered at physiological temperature and pH 7.40 with HEPES/NaOH [20]. The chemical composition of the human blood plasma and SBF is given in Table 1.

#### 2.6.1. Phase Evaluation and Microstructural Characterizations

The prime NFC nanocomposite samples and tested samples in SBF were analyzed using X-ray diffraction (XRD) to find the crystalline phases. The XRD patterns were obtained using a diffractometer (D8 Advance Model, Bruker, Germany) using Cu-k*α* radiation at a wavelength of 1.5 A in the 2*θ* range of 20°-60°. Identification of patterns was performed utilizing JCPDS reference cards and X'Pert HighScore software. The surfaces of samples before and after immersion in SBF were analyzed using scanning electron microscopy (SEM TESCAN-Vega 3, TESCAN, Czech Republic). The samples were coated with a thin layer of gold (Sputtering coating Q150R-ES, Quorum Technologies, England) for better electrical conductivity. The samples were then viewed under the SEM operating at 15 kV. Energy-dispersive spectroscopy (EDS) analysis was performed with a spectrometer (RONTEC, USA) to find elemental distribution through the microstructures.

#### 2.6.2. Fourier Transform Infrared Spectroscopy (FTIR)

To determine the present compounds on the surfaces of samples, FTIR spectra were obtained for NFC samples before and after soaking in SBF using a Tensor II Spectrometer (Germany) and MAUNA-IR 750 equipment. Spectra were acquired in transmission mode in the frequency range of 400 to 4000 cm^−1^.

### 2.7. Cytotoxicity Assessment

The cytotoxicity of NFC was investigated by MTT assay on the periodontal ligament(PDL), according to Abbaszadegan et al. [22]. In this method, 10^6^ cells were suspended in 15 mL of RPMI% medium containing 10% FBS (Fetal Bovine Serum) and transferred by a sampler, 100 *μ*L cell suspension to a 96-well plate, thereby sowing 10^6^ cells. The plate was then incubated in an incubator containing 5% CO_2_ at 37°C to give the cells a stable state. After 24 hours, the culture medium was replaced with the culture medium containing each of the NFC samples at concentrations of 500, 200, 100, 50, 10, and 1 *μ*g/mL, and the plate was returned to the incubator. To the control well (positive control), the cell culture free of NFC was added. The well free of the cell culture was considered as a witness sample (negative control). After 24 hours, the culture medium was discharged and replaced with 30 *μ*L of MTT medium (4 mg/mL). The plate was incubated for 3 hours, and after charging 100 *μ*L of dimethyl sulfoxide (DMSO), it was added to the wells. The plate was shaken for one minute and was incubated for another 10 minutes to dissolve the crystals in DMSO completely. Finally, the absorption of each well was measured by a microplate reader at a wavelength of 570 nm. The cell viability was measured using the following formula [21]:

(2) $$\begin{array}{c} \text{Viability}\left(\%\right)=\frac{A_{\text{t}}-A_{\text{b}}}{A_{\text{c}}-A_{\text{b}}}\times 100, \end{array}$$

where *A*_t_, *A*_b_, and *A*_c_ are absorbance in the test well, absorbance in the witness well, and absorbance in control well, respectively.

### 2.8. Radiopacity

Radiopacity is a crucial physical characteristic that is desired to cement to be identified in the root canal. To evaluate the radiopacity property of the NFC, samples were prepared according to the recommendation of ISO 6876/2012 [17]. To accomplish this, the NFC was compressed into a ring-shaped mold with an internal diameter of 10 mm and a height of 1.0 mm. Then, the samples were kept in an incubator at a temperature of 37°C for 24 hours and under a relative humidity of over 95%. Three specimens of the cement were made for the radiopaque measuring. The samples' radiography was performed along with an aluminum step wedge that was used as a reference. The radiographs were taken using a dental X-ray system (Gilardoni S.P.A, Italy) operating at 60 kV and 4 mA. The exposure time for each examination was 0.3 min, and the target-film distance was 30 cm. A photographic densitometer (Macbeth Model, Italy) was used to control the density of the radiographic images.

### 2.9. Statistical Analysis

In the present study, SPSS IBM subsidized software was used to analyze the data. To evaluate the results of biological experiments, the data was displayed in each group as mean ± standard deviation. A significant difference between mean groups was analyzed by one-way ANOVA analysis and then Tukey test. In all experiments, an average of 3 measurements was used for each group, and a significant level in all statistical tests (*P*) was considered less than 0.05.

## 3. Results

### 3.1. Material Characteristics

**Figure 2:**
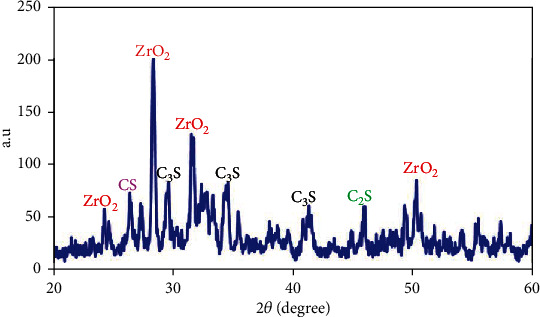
The XRD pattern of the primer powder.

**Figure 3:**
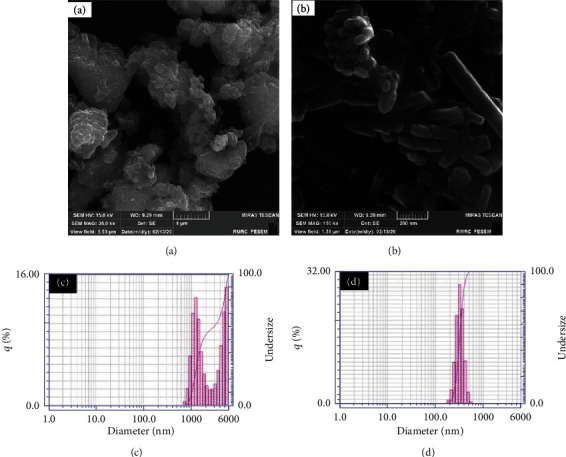
FESM image and particle distribution of the primary cement (a, c), FESM image, and particle distribution of the NFC (b, d).

Phase analysis has been done to find components of the prime cement powders. The XRD patterns of the powders are given in Figure 2. The results indicated that the prime powders consist of dicalcium silicate (C_2_S), tricalcium silicate (C_3_S), and zirconia (ZrO_2_). The FESEM images of primary cement and developed cement powders via the WSMM process (NFC) are shown in Figure 3. Microsized cement powders have been transformed into nanoparticles by WSMM (Figures 3(a) and 3(b)). The average particle size of the primary cement and NFC was measured through the PSA method. The results are presented in Figure 3(c). The PSA analysis indicated that the mean particle size of the primary cement was 2723 nm. The measurements revealed that the cement particle sizes were reduced to 322 nm after WSMM operation (Figure 3(d)).

### 3.2. Setting Behavior of the Cement Pastes

The setting behavior of the cement before milling (primary cement) and after milling (NFC) is presented in Table 2. It can be seen that the final setting time of the cement pastes significantly dropped from 65 to 15 min (more than 4 times reduction) when the particle sizes decreased from 2723 nm to 322 nm.

**Figure 4:**
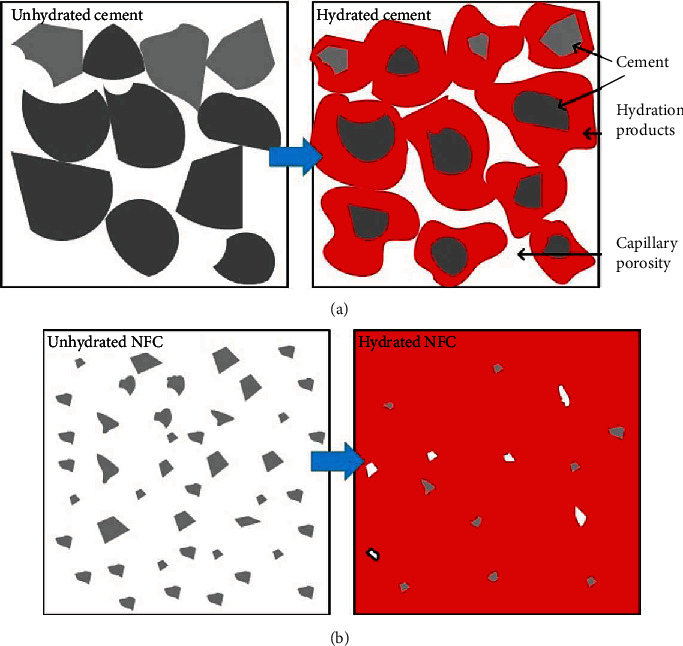
Hydration of primary cement (a) and NFC (b).

The significant reduction in the NFC setting compared to the primary cement is mainly related to the effect of particle size. The particles' fineness produces more surfaces to carry out hydration reactions [23] and causes a higher hydration rate in NFC. The mechanism is modeled in Figure 4.

### 3.3. Evaluation of Color Stability

**Figure 5:**
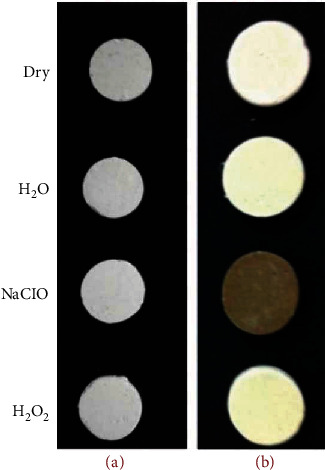
Digital images of (a) NFC and (b) MTA [24] before and after soaking in various solutions.

**Figure 6:**
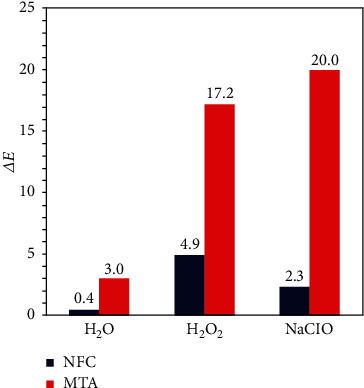
Discoloration of NFC and MTA [24] after soaking in H_2_O, H_2_O_2_, and NaClO.

Evaluation of color stability has been done utilizing visual inspection and spectrometry examination.  Figure 5 displays the digital images of NFC samples before and after soaking in different solutions. The digital images show that the samples' color did not change in distilled water, H_2_O_2_, and NaClO. The color changes of samples were obtained by the spectrometer and are plotted in Figure 6. The results confirmed that the solutions did not cause realizable discoloration on the NFC samples. The spectrometry examination shows that Δ*E* values for NFC are always lower than 5.

### 3.4. In Vitro Bioactivity Test

In vitro bioactivity tests of the nanocement were performed by immersion of NFC samples in SBF at 36.5°C for 0, 1, and 14 days. After soaking in SBF, the development of HA on the surface of nanocement was investigated using phase evaluation, EDX analysis, and microstructural investigation.

#### 3.4.1. Phase Evaluation

**Figure 7:**
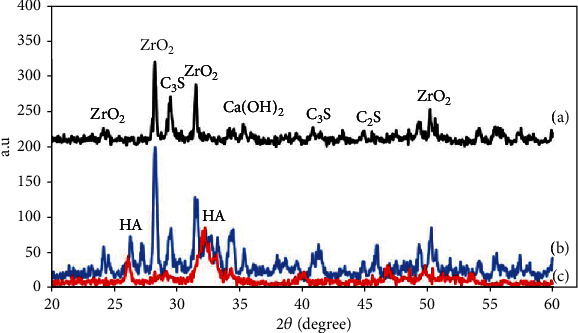
The XRD pattern of (a) NFC before soaking in SBF, (b) NFC after soaking in SBF for 14 days, and (c) pure hydroxyapatite.

Figure 7 shows the XRD results of the hydrated NFC before and after 14-day immersion in SBF. The XRD results of hydrated NFC before immersion in SBF are presented in Figure 7(a). As illustrated, the hydrated NFC consists of di- and tricalcium silicate, calcium hydroxide, and C-S-H gel. Dicalcium silicate and tricalcium silicate, the prime components of the cement that has not been involved in hydration reactions, exhibit strong multipeaks at 29.42°, 32°, 41°, and 46.24°. Calcium hydroxide and C-S-H gel are hydration products. The calcium hydroxide peak is clear at 34.1°. The characteristic peaks of the C-S-H compound are weakly observed at angles of 32.4°, 32.9°, and 46.2°. The C-S-H gel, due to its mainly amorphous structure, exhibits weak peaks in the XRD patterns. The diffraction patterns of zirconium oxide were specified at 2*θ* = 24°, 28°, 31.3, and 50.3°.

The XRD results of hydrated NFC after immersion in SBF are presented in Figure 7(b). The XRD pattern of this sample is similar to that of NFC before immersion in SBF (Figure 7(a)), except that the new peak of hydroxyapatite appears at 2*θ* = 25.8° and 32.1° [25, 26]. The XRD pattern of pure hydroxyapatite (Figure 7(c)) confirms this issue. The hydroxyapatite has a similar composition to bone tissue, and the mechanism of its formation will be explained later.

#### 3.4.2. FTIR Analysis

**Figure 8:**
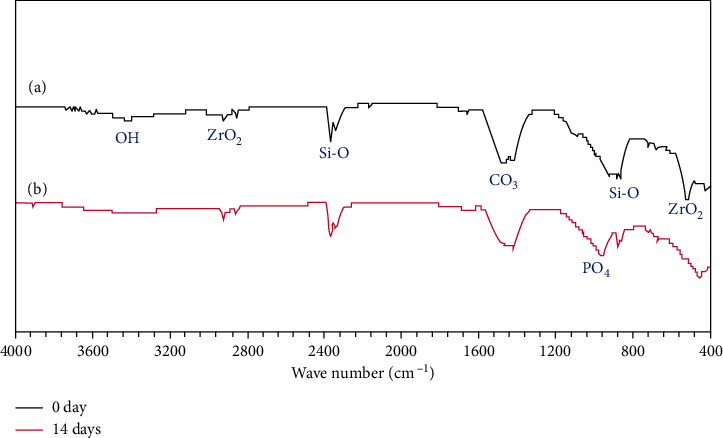
FTIR pattern of the NFC (a) before and (b) after soaking in SBF.

To further detect compounds in the cement and prove the hypothesis that the phase formed on the NFC samples is HA, the infrared Fourier spectroscopy pattern of the NFC before and after soaking in SBF was obtained.  Figure 8(a) shows the spectra of samples before immersion in SBF. The peaks observed at 430, 502, and 2913 cm^−1^ correspond to zirconia. Si-O tensile oscillations caused an adsorption band around 990 cm^−1^ in C-S-H gel, and the peaks around 1488 and 2359 cm^−1^ are related to those oscillations in the carbonate groups. The broad signals around 3430 cm^−1^ are due to H-O oscillations in water and calcium hydroxide.

Figure 8(b) presents the FTIR spectra of the samples after soaking in SBF. In this sample, besides the sample peaks before immersion (Figure 8(a)), bands at about 961 cm^−1^ appear (PO_4_) that may indicate apatite formation on the surface of samples [27, 28]. The results attained utilizing FTIR analysis correspond to those taken by X-ray diffraction.

#### 3.4.3. Microstructural Analysis

**Figure 9:**
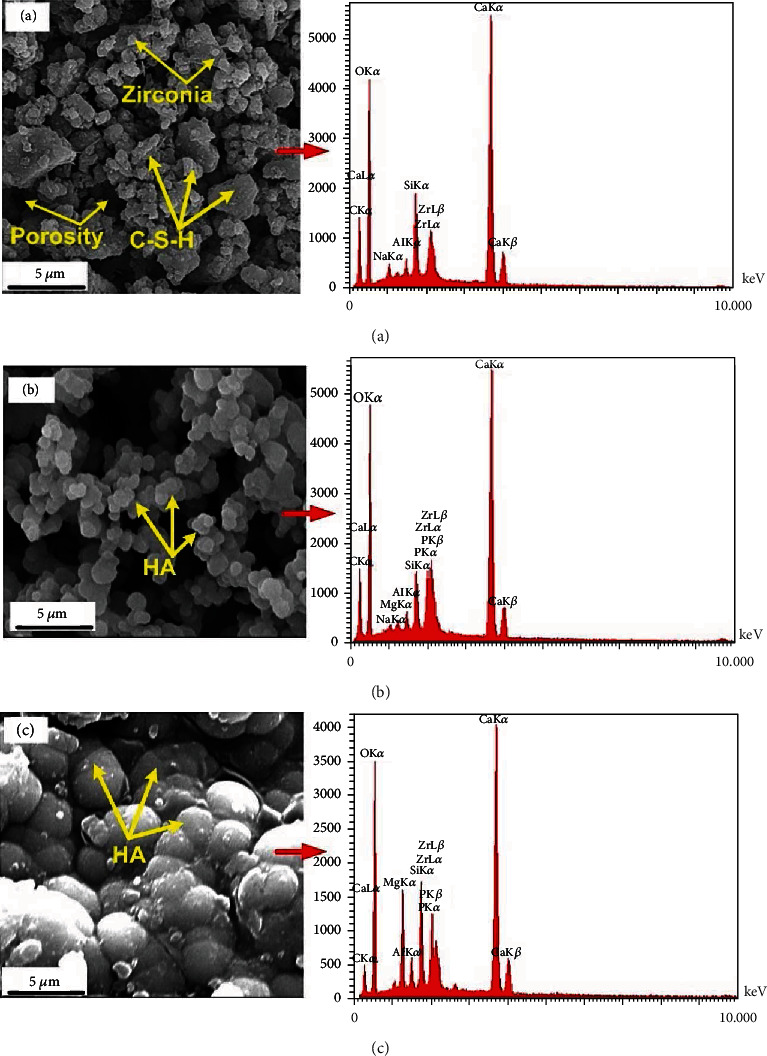
SEM image and EDX analysis of the sample before soaking in SBF (a), after soaking in SBF for 1 day (b), and after soaking in SBF for 14 days (c).

Figure 9 shows the SEM images of setting samples before and after soaking in SBF for 1 and 14 days. The microstructure of the NFC before immersion is presented in Figure 9(a). As shown, the microstructure consists of a C-S-H matrix, calcium hydroxide crystals, and some pores. These components are products of hydration reactions. Zirconia particles interlocking with the hydration products also are observed in the picture.  Figure 9(b) is the SEM image of NFC being immersed in SBF for one day. Nucleation and formation of the apatite on the sample surfaces are visible in the pictures, but it is not significant.  Figure 9(c) presents the microstructure of NFC immersed in the SBF for 14 days. The picture shows that maintaining the NFC for 14 days in SBF led to the development of significant apatite values on the cement surfaces.

#### 3.4.4. EDX Analysis

For more clarification, EDX analysis has been carried out on the cement samples. The results of the elemental EDX analysis of the NFC before and after 1- and 14-day immersion in SBF are shown in Figures 9(a)–9(c). Calcium, silicon, oxygen, and zirconium were found on the samples not immersed in SBF (Figure 9(a)). For the samples immersed in SBF, the results were similar to those of the samples before immersion. The difference is that the elements of calcium and phosphorus were also seen in the EDX analysis (Figures 9(b) and 9(c) for 1- and 14-day immersion, respectively), indicating possible apatite growth. Calcium and phosphorus concentration originating from the SBF increased considerably over time, as is seen in Figures 9(b) and 9(c).

### 3.5. Cytotoxicity

**Figure 10:**
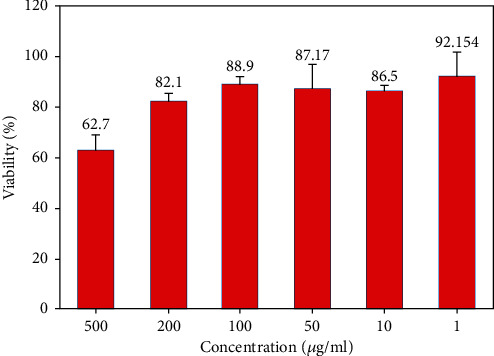
The cytotoxic effects of NFC on MCF-7 cell lines cells under different concentrations.

According to the American Association of Endodontist's recommendations, using a new dental material should be based on biological and clinical principles. An endodontic material must be tested biologically by in vitro examinations before implantation in the body to minimize its detrimental effects. Also, biomaterials that will be in permanent contact with the body tissues should display low cytotoxicity. The MTT assay is a proper method for assessing cell viability and determination of biomaterial toxicity [29]. The process is based on mitochondrial activity and the metabolism of cells. In this work, the proliferation/viability of MCF-7 cells was evaluated by the MTT assay, and the results are presented in Figure 10. These results are for the concentration of the cement nanoparticles in the range of 1–500 *μ*g/mL in wells containing the MCF-7 cells and over 24 hours. Although the viability percentage declined from 92% to about 63% by raising the concentration from 1 to 500 *μ*g/mL, the viability is acceptable. The results revealed that in dilute solution (concentration below 100 *μ*g/mL), the NFC is nontoxic; in moderate concentration (between 100 and 200 *μ*g/mL), the NFC is nearly nontoxic; and in high concentrations (500 *μ*g/mL), the NFC is slightly toxic. The improvement in the viability of cells in low concentrations of NFC may be due to reduced toxic compounds in the cement.

### 3.6. Radiopacity

Radiopacity is a crucial physical characteristic needed in restorative dental materials. A minimal radiopacity value is required for the sealer material to be detected in the root canal and allows the dentist to correct filling failures before final treatment. To understand the quality of zirconium oxide in cement detection in restorative dental radiography, the NFC's radiopacity was obtained. According to the results, the NFC has a radiopacity equivalent to 3.3 mm Al. It is higher than the minimum requirements of dental restorative materials, as stated in ISO 6876/2012 [17].

## 4. Discussion

*Setting behavior*: the setting time of NFC was measured about 15 min that is about 4 times shorter than the prime cement and by about 15 times shorter than MTA that was reported by AlAnezi [8] as is given in Table 2. The considerable reduction in the setting time by decreasing the particle sizes can be explained through the hydration reaction mechanisms. The cement consists of tricalcium silicate and dicalcium silicate that react with water to form a basic solution containing OH^−^, Ca^2+^, and silicate ions [30]. Due to reactions of these anions and cations in the solution, hydrated calcium silicate gel (C-S-H) and calcium hydroxyl form on the surface of cement particles. The reactions can be expressed as [31, 32]

(3) $$\begin{array}{c} {\text{C}}_{3}\text{S}+{\text{H}}_{2}\text{O}⟶\text{C}-\text{S}-\text{H}+\text{Ca}{\left(\text{OH}\right)}_{2}\\ {\text{C}}_{2}\text{S}+{\text{H}}_{2}\text{O}⟶\text{C}-\text{S}-\text{H}+\text{Ca}{\left(\text{OH}\right)}_{2} \end{array}$$

As hydration reactions progress, the hydrated calcium silicate gel polymerizes and converts to a solid network. The gel is relatively impermeable to water, and so it slows down of further reactions [33]. Nanoparticles provide large surface areas and nucleation sites thereby stimulating the hydration reactions [34]. At the same time, the milling processes create more reactive surfaces [34]. This has facilitated the progress and completeness of the hydration reactions as schematically shown in Figure 4.

*Discoloration*: the results of experiments revealed that the NFC is resistant to discoloration in contact with H_2_O, NaClO, and H_2_O_2_ solutions. Comparison of the results with Keskin et al.'s experiments [24] on MTA in similar conditions showed that NFC exhibited higher resistance to color change compared to MTA. The resistance to discoloration is more pronounced when comparing NFC and MTA in contact with NaClO solution. No color change is seen for NFC in contact with NaClO, while it led to severe discoloration of MTA (Figures 5 and 6). The resistance of the NFC to discoloration is related to the use of zirconia instead of bismuth oxide as a contrasting substance. Marciano suggested that discoloration of MTA in the presence of NaClO is due to a change from bismuth oxide to bismuth metal, which is inherently black [35]. It seems zirconia is stable in contact with NaClO and is more suitable for endodontic applications than bismuth oxide. The exact mechanism of its color stability needs further investigation.

*Bioactivity*: the SBF test of the NFC was performed in this investigation for the evaluation of bioactivity behavior. Apatite precipitation on the NFC confirmed by XRD, SEM, and EDX examinations was a strong reason for its proper bioactivity. In explaining this issue, we look at how the body reacts to an external implant. Generally, implantation of a biomaterial in the human body may be confronted with four types of reactions: nearly inert, porous, restorable, and bioactive [36, 37]. In a bioactive material, interfacial bonding of biomaterial and bones is created via ion-exchange processes between the implant and surrounding body fluids [38]. The process causes the development of a biologically active component named apatite on the implant surface, similar to the mineral phase in bone concerning chemical and crystallographic characteristics [39, 40]. The XRD, FTIR, and EDAX examinations detected the formation of the apatite on the specimens. The mechanism's action can be explained as follows:

As soon as NFC is immersed in SBF, a solid-liquid interface is created between the cement and solution, and hydrolysis occurs soon after [41, 42]. Ion exchanges are started between hydration products or C_3_S and C_2_S, where Ca^2+^ from the cement exchange with H^+^ from the aqueous solution form a solid-liquid interface. As a result of the reaction of Ca^2+^ ions with hydroxyl ions derived from water calcium hydroxide or portlandite, the alkaline condition was created. Cation exchange increases OH^−^ concentration of the solution, and so the surfaces of calcium silicate particles were surrounded by the hydroxyl ions of the solution [43]. Another result of hydrolysis is the SiO_4_^4-^ group that in alkaline conditions developed calcium silicate hydrate phase with amorphous microstructure on the surface of the cement [44]. The calcium silicate hydrate is porous water containing silicate gel film consisting of silanol or Si-OH groups. Afterward, the Si-OH group in the calcium silicate hydrate phase loses a proton H^+^ in an alkaline environment, producing a negatively charged surface with the SiO^−^ functional group as [45]

(4) $$\begin{array}{c} \equiv \text{SiOH}+{\text{H}}_{2}\text{O}↔\equiv {\text{SiO}}^{-}+{\text{H}}_{3}{\text{O}}^{+} \end{array}$$

The produced negative functional group attracts the positive calcium ions released in solution under electrostatic interactions, causing the increase of cations on the cement surfaces [46]:

(5) $$\begin{array}{c} \equiv {\text{SiO}}^{-}+{\text{Ca}}^{2+}⟶\equiv {\text{SiO}}^{-}\cdots {\text{Ca}}^{2+} \end{array}$$

It is a double layer with an equal opposite charge, which provided suitable conditions for deposits of other substances. On the other hand, the SBF solution contains PO_4_^3-^ that is hydrolyzed as follows [46]:

(6) $$\begin{array}{c} {\text{H}}_{2}\text{O}+{\text{PO}}_{4}^{3-}⟶\equiv {\text{HPO}}_{4}^{2-}+{\text{OH}}^{-} \end{array}$$

Immersion of calcium silicate in a phosphate-containing solution, SBF, which includes hydrolyzed HPO_4_^2-^, results in electrostatic interactions between HPO_4_^2-^ and Ca^2+^ on the surfaces of the cement, and it produces inhomogeneous nucleation of apatite as [47]

(7) $$\begin{array}{c} \equiv {\text{SiO}}^{-}\cdots {\text{Ca}}^{2+}+{\text{HPO}}_{4}^{2-}⟶\equiv {\text{SiO}}^{-}\cdots {\text{Ca}}^{2+}\cdots {\text{HPO}}_{4}^{2-} \end{array}$$

The formation of apatite on the cement is a reason for its proper adaptation of the NFC in the root canal of the tooth [48, 49]. Good adaptation and sealing capability of the cement sealer play a crucial role in successfully filling the gaps at the cement-dentin interface and preventing the risk of restoration failure causing bacterial microleakage and subsequent clinical problems [50, 51]. The growth of apatite also indicates the high activity of this dental material in the body environment [52] and can be considered as a regenerated material for future investigations.

*Cytotoxicity*: this in vitro study showed that the ability of NFC to induce promotion in cell culture is significant. In this novel material, we replaced bismuth oxide with zirconia as a radiopacifier. Investigations revealed the likely cytotoxic effect of bismuth oxide on the body of osteoblast-like cells while placed aside dicalcium silicate cement [53, 54]. Studies have shown that bismuth oxide also enhances the cytotoxicity in dental pulp cells when added to Portland cement as a radiopacifier. On the other hand, studies have revealed that the body system response to zirconia is nearly neutral. Therefore, the use of zirconia in NFC makes it preferable to MTA concerning its low cytotoxicity.

*Radiopacity*: defects in sealer materials should be well detected on the radiographic films to prevent the saliva from penetrating and causing infection. Radiopacity examination reveals that defects in combination of NFC/zirconia are well recognizable. Zirconium oxide is used as a radiopacifier in NFC contrary to MTA where bismuth oxide is considered for such performance. The idea for such choice might be due to some investigations which revealed that zirconium oxide presents biocompatible behavior and is shown as a bioinert material with desirable corrosion resistance and mechanical properties [55]. Besides, researchers reported evidence of bismuth oxide toxicity aside from calcium silicates, as mentioned earlier [56]. The results of toxicity and biocompatibility tests, as stated in previous sections, showed that zirconia did not cause toxicity aside from NFC.

Consequently, NFC was developed to overcome some deficiencies of the currently available MTA. By the fineness of the cement particles at the nanoscale (NFC), the setting time was reduced 12 times compared to MTA. Another problem associated with MTA was reported as discoloration. In this investigation, bismuth oxide, which was reported as the main cause of the color change in the presence of MTA, is replaced by zirconia. The results of discoloration indicated that NFC presents a high resistance against solutions such as H_2_O, NaClO, and H_2_O_2_ and is another advantage of this material over MTA. Additionally, other characteristics of endodontic material must be bioactivity, biocompatibility, and nontoxicity. Nanofast cement showed proper bioactive behavior and nontoxic characteristics in all ranges of concentration. Endodontic sealer cement also must show proper radiopacity to allow the quality of restoration to be radiographically visualized. NFC in the presence of zirconia showed adequate radiopacity by the recommendation of ISO 6876/2012.

## 5. Conclusion

The present work deals with setting behavior, color stability, bioactivity, cytotoxicity, and radiopacity of a new calcium silicate base cement named NFC. NFC consisted of the di- and tricalcium silicate (C_2_S and C_3_S), as the base material, and zirconium oxide, as a radiopacifier, in which their particle size has been reduced to nanosize via WSMM processes. The final setting time of the developed cement considerably reduced from 65 to 15 min by reducing particle sizes from 2723 nm to 322 nm. The discoloration of NFC was investigated via spectroscopy examination. It was found that the combination of NFC/zirconia represents acceptable discoloration. The phase evaluation and FTIR examination revealed the formation of hydroxyapatite on the soaked samples in SBF for 1 and 14 days. The hydroxyapatite was also detected in microstructural observations. This is evidence of the favorable bioactivity and biocompatibility of the NFC. The cytotoxicity of NFC was investigated by MTT assay on the MCF-7 cell line. NFC showed a positive biological property and may be a safe choice for restoration treatment. The radiopacity of the NFC/zirconia dental material was examined using a radiographer. It was found that zirconia provides a radiopacity corresponding to 3.3 mm/Al and meets the standard requirements.

## Figures and Tables

**Table 1:** Ion concentration (mM) in simulated body fluid in comparison with blood plasma [21].

Formulation	Na^+^	K^+^	Ca^2+^	Mg2^+^	Cl^−^	HCO^3-^	HPO_4_^2-^	SO_4_^2-^
Human blood plasma	142	5	2.5	1.5	103	27	1	0.5
SBF	142	5	2.5	1.5	147.8	4.2	1	0.5

**Table 2:** Comparison of setting time of the primary cement, NFC, and MTA.

Material	Initial setting time (min)	Final setting time (min)
Primary cement	10	65
NFC	5	15
MTA [8]	40	175

## Data Availability

Data is available on request.
